# Mothercraft Hostels

**Published:** 1920-01-24

**Authors:** 


### Mothercraft Hostels.

A SCHEME to help the unmarried mother was described in the *Daily Chronicle* last week in the words of a trained nurse who is shortly to open a hostel in Liverpool. She considered the bad point in all rescue work had been the separation of mother and child, and expressed her opinion that most social workers to-day believe it best for the child and mother to be together.

"The object (said the nurse) of the Liverpool scheme, which will be run under a committee of interested people, is to provide a hostel for unmarried expectant mothers where their babies can be born. It is important that unmarried mothers should be taught how to care for their babies, because the death-rate among illegitimate children is high. As far as I know few hostels where these babies are received give the mothers a really adequate training in mothercraft.

"We shall insist that, whenever possible, the mother shall feed her child, and she will not be expected to go out to work until the baby is at least six months old.

"During this time, if she has not already a trade or profession, she will be taught how to earn her own living, and, when able to leave her baby, she will go out to daily work, and come back to the hostel at night."

There is a home in London run on similar lines—the Day Servants' Hostel, in Danvers Street, Chelsea—where there are sixteen unmarried mothers who go out to work every day and return to their babies when work is done. The superintendent says the aim of the hostel is to create a home atmosphere of a genuine type, where mothers and babies can live happily and normally. She hopes that the last [illegible] been seen of schemes for big institutions.

